# Food Faddists and Something Worse

**Published:** 1915-08-14

**Authors:** 


### THE EDITOR'S LETTER-BOX.

### Food Faddists and Something Worse.

*To the Editor of* THE HOSPITAL.

SIR,—While noting with approval your criticism of the food faddist in an Editorial Note appended to the letter on "Economy and the Food Question" in THE HOSPITAL last week, may I be permitted to point out that there is someone even worse than the food faddist, and that is the food-faker, who attempts to foist on our wounded men, by exploiting the sympathies of their relatives, his own patent pepsins and peptones in the guise of packets of nourishing foods for soldiers on active service?

Of course commercialism will have its fling, but relatives, not always the most tactful people of our acquaintance, would be surprised, I am credibly informed, at the reception which these packets of doubtless valuable, but generally unappetising, vegetarian and other concentrated "foods" receive on being opened by a hungry man in the trench or on the march.

Some established firms, whose names are known to every one of your readers, have reputation and a name which guarantees the scientific trustworthiness of their lozenges and compressed foods, but the present demand for presents of "luxuries" for our fine fellows at the Front is being exploited by less scrupulous salesmen, and I think the matter is worthy of your notice.—Yours faithfully, HOSPITAL.

[If our correspondent will turn to page 421 he will find an article on "Frauds on German Soldiers," which, quoted on American medical authority, points to the conclusion that German efficiency, organisation, and business sense have produced scientific food-fakers of a class which our worst faddists at home can hardly hope to rival. These seem to agree with the classic economist of the Manchester School, who remarked, if we remember, that adulteration was the purest form of competition.—ED. THE HOSPITAL.]

### Combined Coal and Gas Kitchener.

*To the Editor of* THE HOSPITAL:

